# Supplementary material for: Combinatorial expression of neurexins and LAR-type phosphotyrosine phosphatase receptors instructs assembly of a cerebellar circuit
Source: Nat Commun. 2023 Aug 17;14:4976. doi: 10.1038/s41467-023-40526-0 (PMC10435579; doi:10.1038/s41467-023-40526-0)
Supplement: Supplementary file 1 — Supplementary Information [file 41467_2023_40526_MOESM1_ESM.pdf]

SUPPLEMENTARY FIGURES and FIGURE LEGENDS

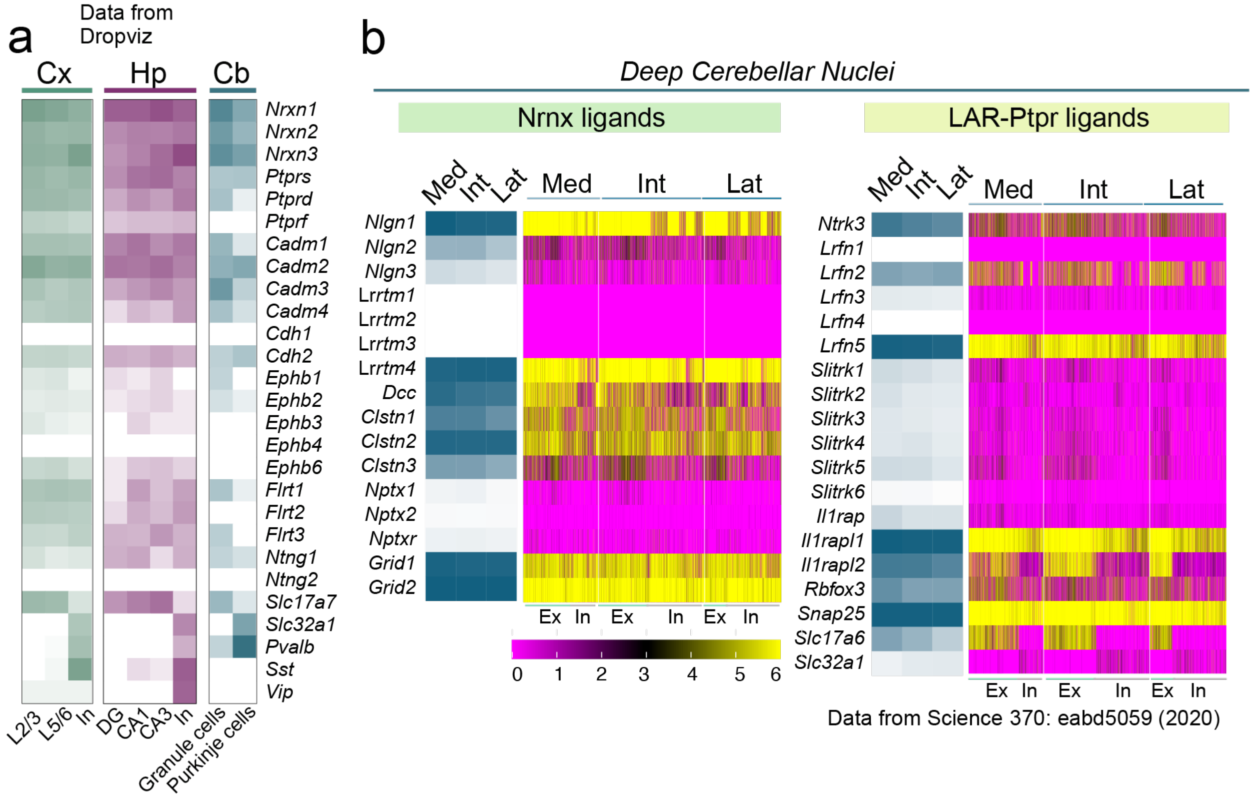

Figure S1 | Expression patterns of neurexins and LAR-PTPRs in mouse brain

a, Summary heatmaps for the expression of presynaptic adhesion molecules (SAMs) in cortical, hippocampal, and cerebellar neurons. Data were downloaded from the Dropviz website (McCaroll laboratory).

b, Summary and single cell heatmaps for Neurexin and LAR-Ptpr postsynaptic ligands in the medial, interposed, and lateral deep cerebellar nuclei. Data were obtained from[62].

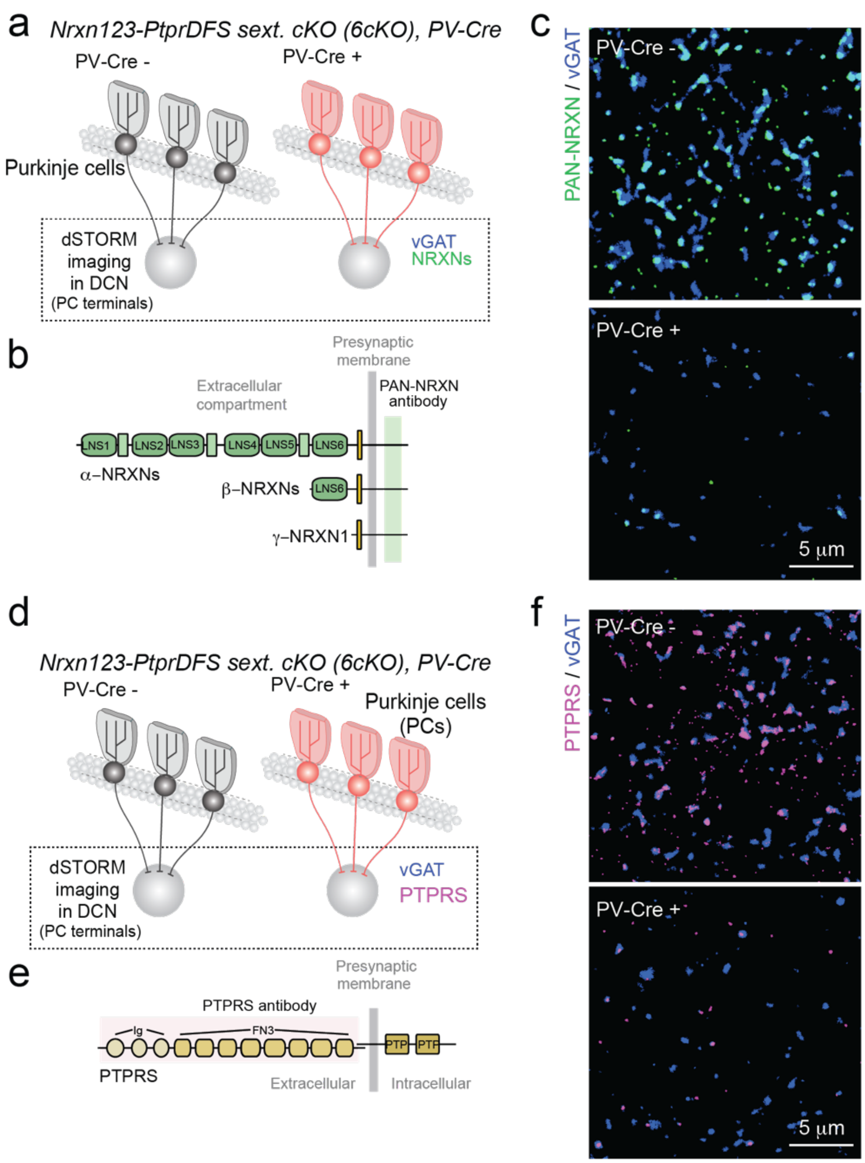

Figure S2 | Characterization of neurexin and PtprS antibodies

a-c, Validation for the anti-PAN Nrxn antibody. **a,** Experimental design for dSTORM imaging in DCN slices; **b,** schematic of the epitope recognized by the antibody; **c,** representative images for Neurexin (green, 647) and Purkinje cell boutons in the DCN (visualized with antibodies to vGAT, blue, 568). Images are representative for 5ROIs/ 1 mouse.

d-f, Validation for the anti-PTPRS antibody. **d,** Experimental design; **e,** schematic of the epitope recognized by the antibody; **f,** representative images for PTPRS (magenta, 647) and Purkinje cell boutons in the DCN (visualized with antibodies to vGAT, blue, 568). Images are representative for 5ROIs/ 1 mouse.

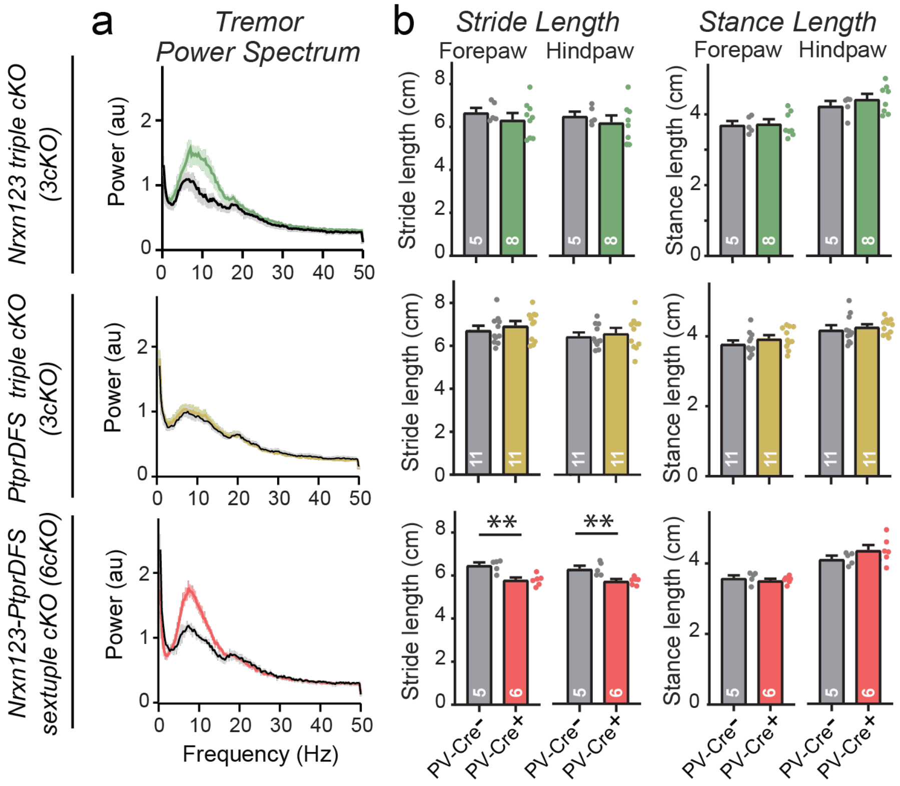

Figure S3 | Further data on the motor behavior of Nrxn123 and PtprDFS triple and sextuple cKO mice

a, Averaged power spectra of tremor behavior at different frequencies, analyzed using measurements from a 10 min force-plate assay, related to Figure **2b-c**.

b, Stride length, stance length, and paw base length for *Nrxn123* 3cKO (top), *PtprDFS* 3cKO (middle), and *Nrxn123-PtprDFS* 6cKO (bottom), related to Figure **2d**.

All data in summary graphs are means ± SEM. Statistical significance was assessed by two-tailed Mann-Whitney test for summary graphs in **b**. For **b**, n=mice: for Nrxn123 3cKO mice (PV-Cre- n=5, PV-Cre+ n=8); for PtprDFS 3cKO (PV-Cre- n=11, PV-Cre+ n=11); for Nrxn123, PtprDFS 3cKO (PV-Cre- n=5, PV-Cre+ n=6). Source data and statistical results are provided within the Source Data file.

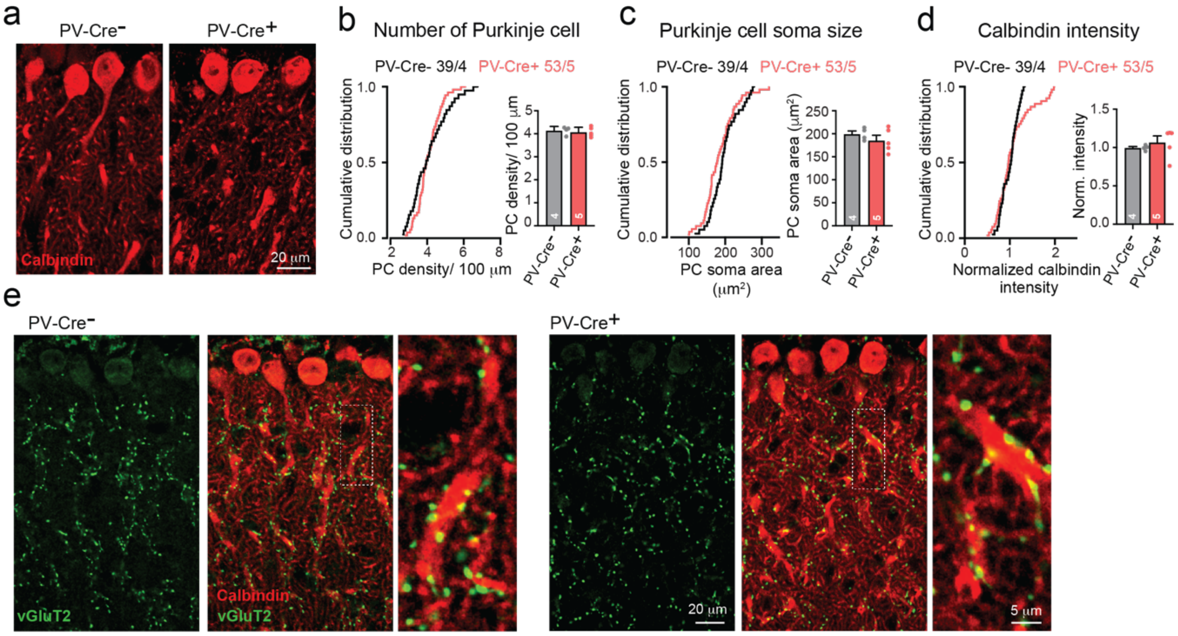

Figure S4 | Detailed analyses of Purkinje cells as a function of the sextuple Nrxn123-PtprDFS deletion

a, Representative images of Purkinje cells from PV-Cre- and PV-Cre+ Nrxn123-PtprDFS 6cKO littermates, stained with Calbindin (red).

b, Cumulative plots of individual data points (n= ROIs/mice) and summary graphs of the density of Purkinje cells in the cerebellum from PV-Cre- and PV-Cre+ Nrxn123-PtprDFS 6cKO littermates.

c-d, Cumulative plots of individual data points (n= ROIs/mice) and summary graphs of the soma size of Purkinje cells (**c**) and Calbindin intensity (**d**).

e, Representative confocal images of Purkinje cells immunostained with antibodies to Calbindin (red) and vGluT2 (green) as a marker for climbing-fiber synapses. Images referred to Figure **3f-h** and are representative for 45 ROIs/4 mice (PV-Cre-) and 53 ROIs /5 mice (PV-Cre+).

All data in summary graphs are means ± SEM. Statistical significance was assessed by Kolmogorov-Smirnov test for cumulative plots and two-tailed Mann-Whitney test for summary graphs. For **b-d**, n= ROI/mouse (PV-Cre- n= 39/4; n=53/5). Source data and statistical results are provided within the Source Data file.

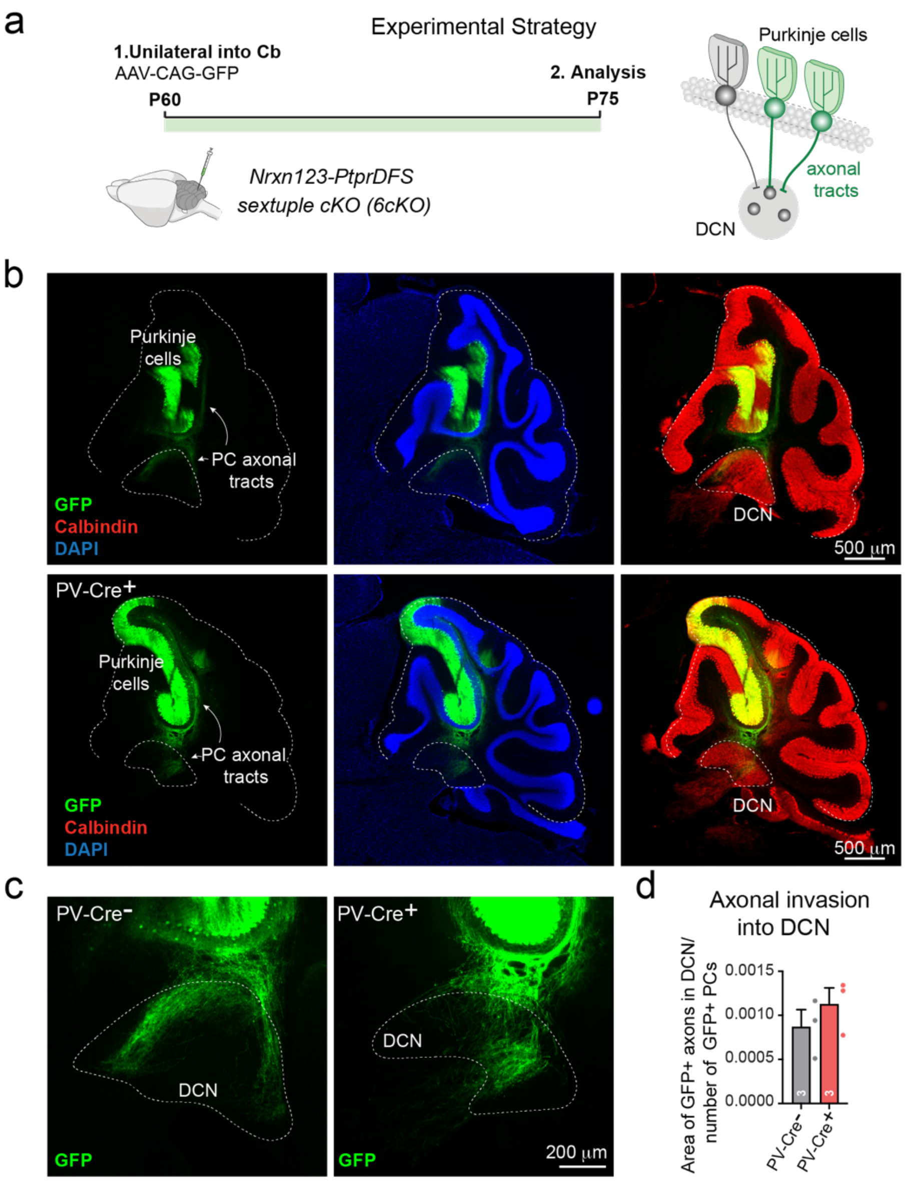

Figure S5 | Anterograde tracing of Purkinje cell axons into DCN in Nrxn123-PtprDFS 6cKO mice does not uncover a major change in axon growth

a, Experimental strategy for **b-c**. AAVs encoding for GFP under the control of the CAG promoter were injected in the cerebellum to allow tracing of PC axons.

b, Representative images of the cerebellum from PV-Cre- and PV-Cre+ Nrxn123-PtprDFS 6cKO littermates, stained for Dapi (blue) and Calbindin (red). Purkinje cells labeled with AAV-CAG-GFP, and their axonal tracts are shown in green (note that AAVs efficiently infect Purkinje cells, while only sparsely target granule cells. Granule cell labeling does not affect tracing of PC axons, as their projections do not overlap with PC).

c, Higher magnification for **b.**

d, Quantification of PC axonal infiltration in the DCN, calculated by normalizing the fraction of the area occupied by GFP-positive PC axons in the DCN to the number of GFP-positive PC cells.

All data in summary graphs are means ± SEM. Statistical significance was assessed by two-tailed Mann-Whitney. For **d**, n=mice: Nrxn123-PtprDFS 6cKO littermates (PV-Cre- n= 3 mice, PV-Cre+ n= 3 mice). Source data and statistical results are provided within the Source Data file.

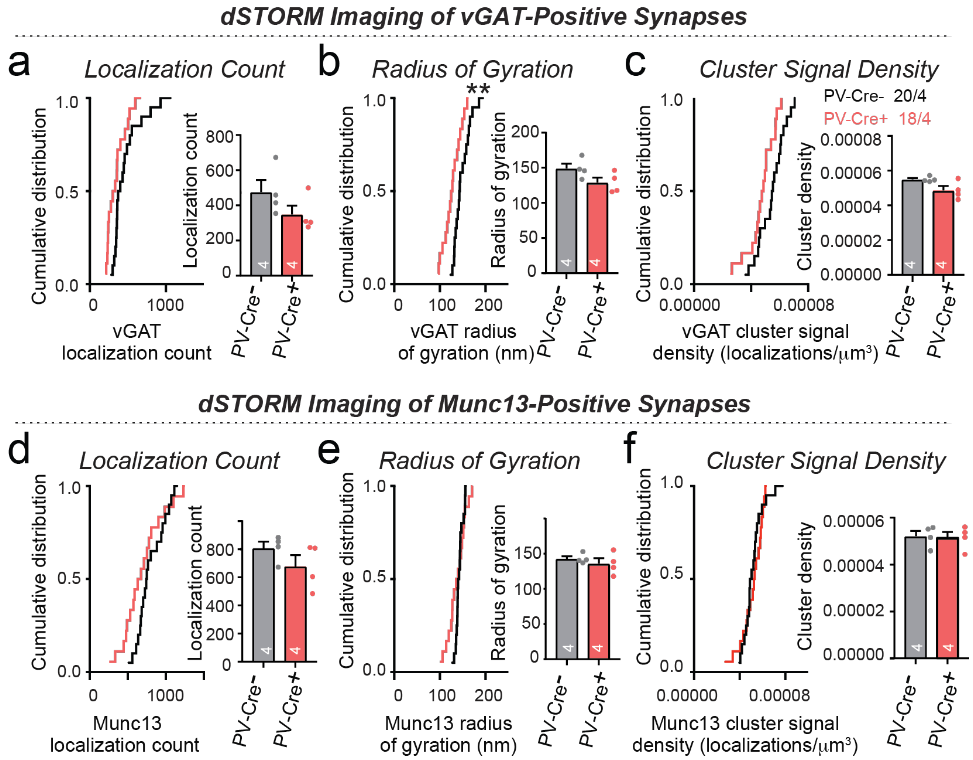

Figure S6 | Further analysis of presynaptic vGAT and Munc13 clusters visualized by dSTORM

a-c, Cumulative plots and summary graphs illustrating the properties of vGAT in the DCN of Nrxn123-PtprDFS sextuple cKO mice.

d-f, Cumulative plots and summary graphs illustrating the properties of Munc13.

All data in summary graphs are means ± SEM. Statistical significance was assessed by Kolmogorov-Smirnov test for cumulative plots and two-tailed Mann-Whitney test for summary graphs. For **a-f**, n=ROI/mice: Nrxn123, PtprDFS 6cKO mice (n= PV-Cre- n=20/4, PV-Cre+ n=18/4). Source data and statistical results are provided within the Source Data file.

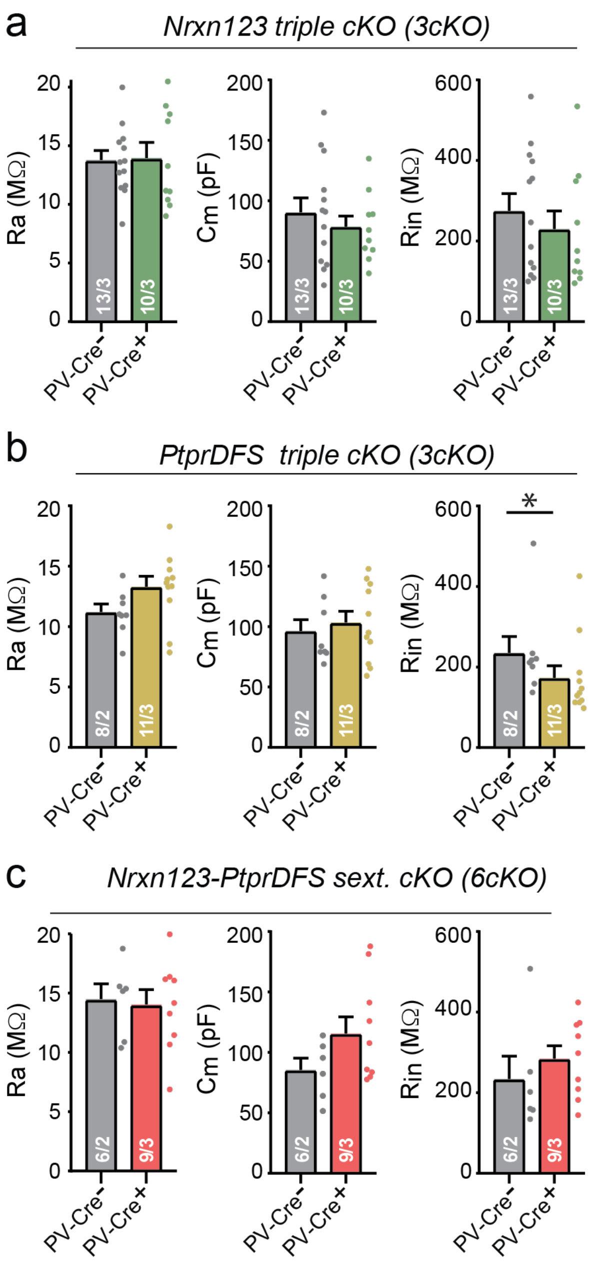

Figure S7 | Passive electrical properties of DCN neurons as a function of the triple and sextuple Nrxn123 and PtprDFS deletions

a-c, Access resistance (Ra), capacitance (Cm) and input resistance (Rin) for DCN neurons from *Nrxn123* 3cKO (**a**), *PtprDFS* 3cKO (**b**), and *Nrxn123-PtprDFS* 6cKO (**c**) mice, measured during electrophysiological recordings.

All data in summary graphs and plots are means ± SEM. Statistical significance was assessed by two-tailed Mann-Whitney for summary graphs. For **a-c**, n= cells/mice: Nrxn123 3cKO mice (PV-Cre- n= 13/3, PV-Cre+ n= 10/3); PtprDFS 3cKO mice (PV-Cre- n= 8/2, PV-Cre+ n= 11/3); Nrxn123, PtprDFS 6cKO mice (PV-Cre- n= 6/2, PV-Cre+ n= 9/3). Source data and statistical results are provided within the Source Data file.
