# Supplementary material for: Combinatorial expression of neurexins and LAR-type phosphotyrosine phosphatase receptors instructs assembly of a cerebellar circuit
Source: Nat Commun. 2023 Aug 17;14:4976. doi: 10.1038/s41467-023-40526-0 (PMC10435579; doi:10.1038/s41467-023-40526-0)
Supplement: Supplementary file 3 — Reporting Summary [file 41467_2023_40526_MOESM3_ESM.pdf]

nature portfolio

Corresponding author(s): Sclip A.

Last updated by author(s): 5/26/2023

Reporting Summary

Nature Portfolio wishes to improve the reproducibility of the work that we publish. This form provides structure for consistency and transparency in reporting. For further information on Nature Portfolio policies, see our Editorial Policies and the Editorial Policy Checklist.

Statistics

For all statistical analyses, confirm that the following items are present in the figure legend, table legend, main text, or Methods section.

n/a	Confirmed	
☐	☒	The exact sample size (*n*) for each experimental group/condition, given as a discrete number and unit of measurement
☐	☒	A statement on whether measurements were taken from distinct samples or whether the same sample was measured repeatedly
☐	☒	The statistical test(s) used AND whether they are one- or two-sided<br>*Only common tests should be described solely by name; describe more complex techniques in the Methods section.*
☐	☒	A description of all covariates tested
☐	☒	A description of any assumptions or corrections, such as tests of normality and adjustment for multiple comparisons
☐	☒	A full description of the statistical parameters including central tendency (e.g. means) or other basic estimates (e.g. regression coefficient) AND variation (e.g. standard deviation) or associated estimates of uncertainty (e.g. confidence intervals)
☐	☒	For null hypothesis testing, the test statistic (e.g. *F*, *t*, *r*) with confidence intervals, effect sizes, degrees of freedom and *P* value noted<br>*Give P values as exact values whenever suitable.*
☒	☐	For Bayesian analysis, information on the choice of priors and Markov chain Monte Carlo settings
☒	☐	For hierarchical and complex designs, identification of the appropriate level for tests and full reporting of outcomes
☒	☐	Estimates of effect sizes (e.g. Cohen's *d*, Pearson's *r*), indicating how they were calculated

Our web collection on statistics for biologists contains articles on many of the points above.

Software and code

Policy information about availability of computer code

Data collection	For patch clamp recordings Clampex 10.7 (Molecular Device) was used. Confocal images were acquired with the Nikon A1RSi microscope. dSTORM imaging was performed on a Vutara SR352 microscope
Data analysis	Clampfit 10.7 and Igor Pro 7.08 were used for analysis of all electrophysiological data. Confocal images were analyzed with the NIS elements software provided by Nikon. dSTORM images were analyzed on the Vutara SRX software (version 6.04). Statistical analysis was done using GraphPad Prism (version 9).

For manuscripts utilizing custom algorithms or software that are central to the research but not yet described in published literature, software must be made available to editors and reviewers. We strongly encourage code deposition in a community repository (e.g. GitHub). See the Nature Portfolio guidelines for submitting code & software for further information.

Data

Policy information about availability of data

All manuscripts must include a data availability statement. This statement should provide the following information, where applicable:

- Accession codes, unique identifiers, or web links for publicly available datasets
- A description of any restrictions on data availability
- For clinical datasets or third party data, please ensure that the statement adheres to our policy

All relevant data supporting the findings of this study are available. Source data have been provided. Databases used for this study include dropviz.org (supplementary figure 1a) and data from NCBI Gene Expression Omnibus under accession GSE160471 (supplementary figure 1b).

Human research participants

Policy information about studies involving human research participants and Sex and Gender in Research.

Reporting on sex and gender	N/A
Population characteristics	N/A
Recruitment	N/A
Ethics oversight	N/A

Note that full information on the approval of the study protocol must also be provided in the manuscript.

Field-specific reporting

Please select the one below that is the best fit for your research. If you are not sure, read the appropriate sections before making your selection.

☒ Life sciences ☐ Behavioural & social sciences ☐ Ecological, evolutionary & environmental sciences

For a reference copy of the document with all sections, see nature.com/documents/nr-reporting-summary-flat.pdf

Life sciences study design

All studies must disclose on these points even when the disclosure is negative.

Sample size	Sample sizes are indicated in the figure legends and/or figures, and were determined based on historical practices in the lab (Sclip, Elife, 2020; Chen et al.,Neuron, 2017; Luo et al., Nature Communications, 2021) to claim statistical effects. Careful effort was made when possible to average per true biological replicate rather than use pseudo-replicates. We did not conduct a power analysis or employ other statistical methods to predetermine sample size.
Data exclusions	No data were excluded from the analysis
Replication	Effort was made to incorporate variation by sampling from different litters. Key phenotypes were reproduced across different measurement types (behavior, electrophysiology, immunostaining and superresolution). Experiments in the paper underwent at least 3 independent replications. Images in figure 1 and supplementary figure 2 were representative of 5 ROIs/1 mouse, or 10 ROIs/1 mouse as indicated in the figure and figure legend). Number of pseudoreplicates (i.e. n=ROIs) and true replicates (n=mice) are reported in each figure/ figure legend.
Randomization	Mice were randomly assigned to groups for each experiments.
Blinding	All experiments were performed blindly by the experimenters.

Reporting for specific materials, systems and methods

We require information from authors about some types of materials, experimental systems and methods used in many studies. Here, indicate whether each material, system or method listed is relevant to your study. If you are not sure if a list item applies to your research, read the appropriate section before selecting a response.

Materials & experimental systems

n/a	Involved in the study	
	x	Antibodies
	x	Eukaryotic cell lines
x		Palaeontology and archaeology
	x	Animals and other organisms
x		Clinical data
x		Dual use research of concern

Methods

n/a	Involved in the study	
x		ChIP-seq
x		Flow cytometry
x		MRI-based neuroimaging

Antibodies

Antibodies used

PAN-NRXN antibody (ABN161-I, Millipore Sigma, 1:300)
PTPRS (PAC9986, homemade, 1:300)
vGAT (131004, Synaptic Systems, 1:1000)
MUNC-13 (126103, Synaptic Systems, 1:1000)
vGluT1 (AB5905, Millipore Sigma, 1:1000)
vGluT2 (AB2251-I, Millipore Sigma, 1:1,000)
Calbindin (C9848, Millipore Sigma,1:1000)

Goat anti-Mouse IgG CF568 (Biotium, 20100-1 mg; 1:3000, IHC)
Goat anti-Rabbit IgG Alexa647 (ThermoFisher, A-21245; 1:1000-1:3000, IHC)
Goat anti-Mouse IgG Alexa546 (ThermoFisher, A11003; 1:1000, IHC)
Goat anti-Guinea Pig IgG Alexa488 (ThermoFisher, A-11073; 1:1000, IHC)
Goat anti-Rabbit IgG Alexa488 (ThermoFisher, A-11008; 1:1000, IHC)

Validation

-PTPRS and PAN-Nrxn were previously validated using KO mice (Sclip et. al, 2020; Trotter et al., 2019) and further validated here (supplementary figure 2).
-vGAT (131004, Synaptic Systems, 1:1000) was validated by the vendor in knockout mice.
-MUNC-13 (126103, Synaptic Systems, 1:1000) was validated by the vendor in knockout mice (PubMed: 28772123)
-vGluT1 (AB5905, Millipore Sigma, 1:1000) was validated by the vendor and previously used to label parallel fiber synapses in the laboratory (Liu et al., Elife, 2022)
-vGluT2 (AB2251-I, Millipore Sigma, 1:1,000) was validated by the vendor and previously used to label climbing fiber synapses in the laboratory (Chen et al., Neuron, 2017; Liu et al., Elife, 2022)
-Calbindin (C9848, Millipore Sigma,1:1000) is a marker for Purkinje cells.
-All secondaries extensively validated by vendors, previous papers from lab, and often test with omission of primary antibodies during initial optimization of staining conditions.

Eukaryotic cell lines

Policy information about cell lines and Sex and Gender in Research

Cell line source(s)	HEK293T, directly purchased from ATCC
Authentication	The cell line was not authenticated (other than by morphology and passage).
Mycoplasma contamination	Cell lines were tested negative for mycoplasma contamination using the fluorochrome Hoechst DNA stain and the direct culture method.
Commonly misidentified lines (See ICLAC register)	No commonly misidentified lines were used.

Animals and other research organisms

Policy information about studies involving animals; ARRIVE guidelines recommended for reporting animal research, and Sex and Gender in Research

Laboratory animals

Experimental mice included both males and females between 20-80days. The following strains were used:
-PV-Cre mice (Jax, stock 017320)
-L7-Cre mice (Jax, stock 004146)
-RiboTag mice (Jax, stock 029977)

	-C57BL6/6J mice (Jax, stock 000664)<br>-Nrxn123 triple cKO mice (Chen et al., Neuron, 2017) were crossed to PV-Cre lines.<br>-LAR-PtprDFS triple cKO mice (Sclip et al., Elife, 2020) were crossed to PV-Cre lines.<br>-Nrxn123,LAR-PtprDFS sextuple cKO mice (generated by crossing Nrxn and Lar-Ptpr triple cKO mice) were crossed to PV-Cre lines.<br>All mice were housed in groups of 2 to 5 on a 12h light/dark cycle with access to food and water ad libidum. Rooms were maintained with 40–60% humidity and at approximately 22°C. All procedures conformed to National Institutes of Health Guidelines for the Care and Use of Laboratory Mice and were approved by the Stanford Animal Use Committees [Administrative Panel for Laboratory Animal Care (APLAC/) Institutional Animal Care and Use Committee (IACUC)].
Wild animals	No wild animals were used in this study
Reporting on sex	Both males and females were used in this study
Field-collected samples	This study did not involve field collected samples
Ethics oversight	All procedures conformed to National Institutes of Health Guidelines for the Care and Use of Laboratory Mice and were approved by the Stanford Animal Use Committees [Administrative Panel for Laboratory Animal Care (APLAC/) Institutional Animal Care and Use Committee (IACUC)]

Note that full information on the approval of the study protocol must also be provided in the manuscript.
